# Heart Transplantation in a Toddler with Cardiac Kawasaki Disease

**DOI:** 10.3389/fsurg.2017.00021

**Published:** 2017-04-20

**Authors:** Theodor Tirilomis, Michael Steinmetz, Marius Grossmann, Anselm Bräuer, Thomas Paul, Wolfgang Ruschewski, Friedrich A. Schöndube

**Affiliations:** ^1^Department of Thoracic, Cardiac, and Vascular Surgery, University of Göttingen, Göttingen, Germany; ^2^Department of Pediatric Cardiology, University of Göttingen, Göttingen, Germany; ^3^Department of Anesthesiology, University of Göttingen, Göttingen, Germany

**Keywords:** Kawasaki disease, coronary artery, coronary aneurysm, myocardial infarction, ischemia, heart transplantation

## Abstract

Kawasaki disease is very rare in Western Europe. The disease may involve coronary arteries. A 2-year-old boy diagnosed with Kawasaki disease had had seizure-like symptoms. Further evaluation revealed recurrent myocardial ischemia and myocardial infarction. Due to extraordinary extension of the coronary disease, myocardial revascularization was not feasible and the toddler underwent successful heart transplantation after 97 days on waiting list.

## Introduction

**Figure 1:**
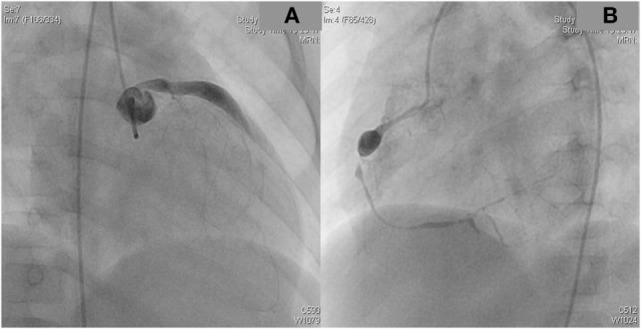
**The coronary angiogram demonstrated proximal aneurysms and the extensive stenotic changes of the left (A) and the right coronary artery (B) caused by Kawasaki disease**.

**Figure 2:**
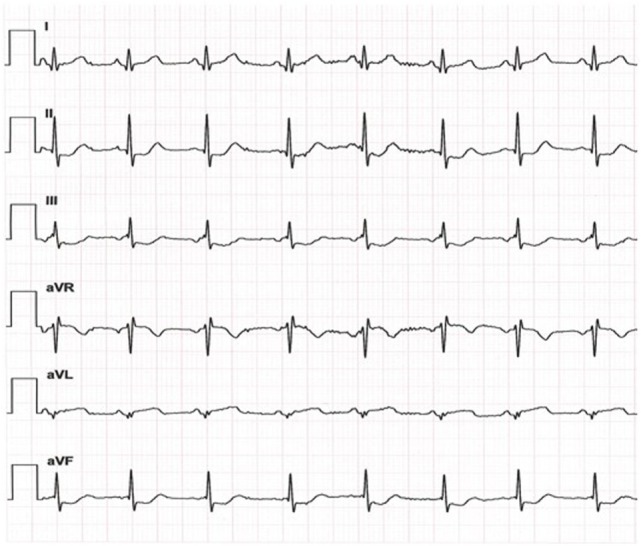
**While waiting for heart transplantation, recurrent ischemic heart attacks were observed; exemplary electrocardiogram (day 14 on waiting list) showing significant ST depression in lead II, III, and aVF**.

**Figure 3:**
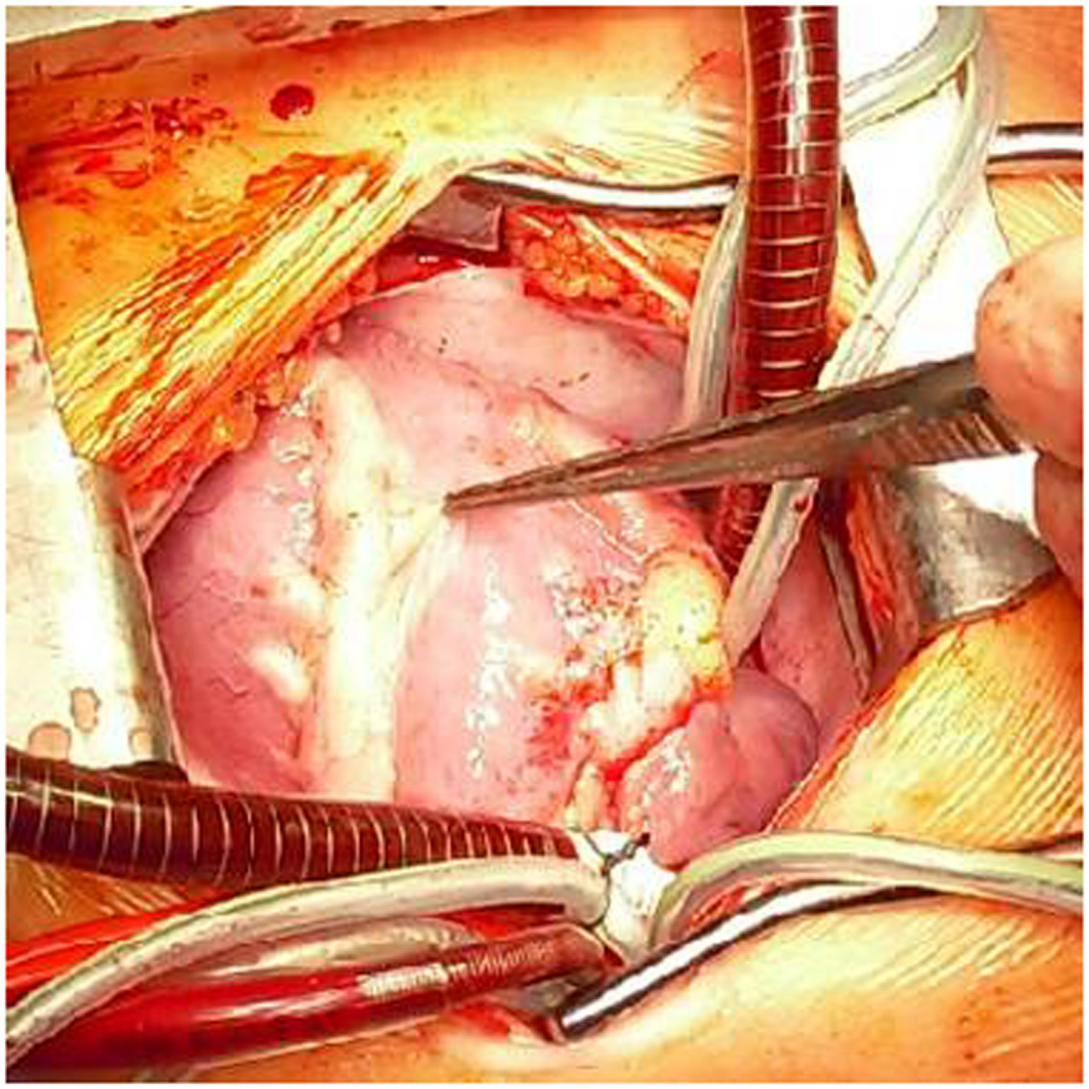
**Intra-operative view of the diseased heart with the extensive changes of the coronary arteries caused by Kawasaki disease**. In front is the left anterior descending artery visible.

**Figure 4:**
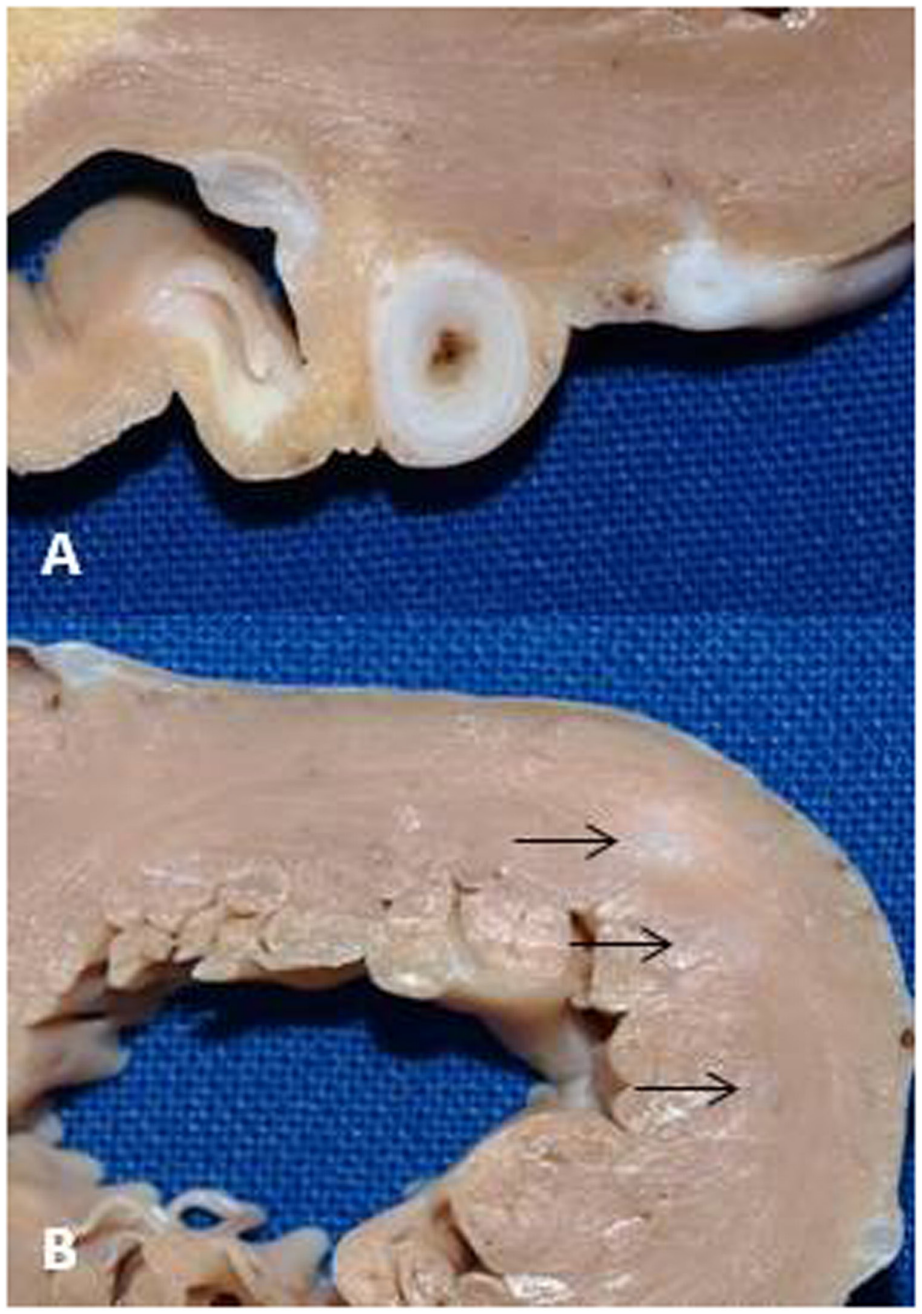
**(A)** Cross-section of the proximal region of the left anterior descending artery showing the excessive thickening of the coronary artery wall at the level of aneurysm. **(B)** Multiple infarctions were presented in the left ventricular wall (arrows).

A 2-year-old boy diagnosed with Kawasaki disease was initially treated with immunoglobulines and abciximab. About 4 months after the diagnosis, the boy suffered a seizure-like episode at home while he was on aspirin, clopidogrel, and phenprocoumon. After admission to the hospital, neurological findings were normal but Holter electrocardiogram revealed repeated periods of significant ST depression during the registration period. Additionally, ST elevation was observed on the patient monitoring. Therefore, coronary angiography was performed. The angiogram revealed coronary aneurysms (6 mm in diameter), but the more important finding was the severity and the extent of stenotic lesions of all coronary arteries (Figure 1). Due to the lack of a promising treatment (conservative, interventional, or surgical), the child was evaluated for heart transplantation and then placed on the waiting list. Due to increased risk of refractory arrhythmias or cardiac decompensation, the child remained under continuous monitoring in pediatric intensive care unit (PICU) and an extracorporeal life support device was ready for use if needed. While waiting for transplantation, many ischemic episodes were documented (Figure 2). Finally, after 97 days on the waiting list, the boy underwent successful heart transplantation. The diseased heart showed severe changes of the coronary arteries. The coronary arteries resembled “chicken feet”: thick, fibrotic, and sclerotic with proximal aneurysms (Figure 3). Transsections of explanted heart revealed severe thickening of the coronary arterial wall and multiple myocardial infarctions (Figure 4). After transplantation, we started immunosuppression with prednisolone, mycophenolate mofetil, and tacrolimus. We did not use induction therapy. Perioperative antibiosis consisted of cefotaxime and vancomycin. The first endomyocardial biopsy performed 1 week after transplantation because of pericardial effusion was without signs of rejection (ISHLT grade 0R). The postoperative course was eventful, and the toddler was discharged from hospital on postoperative day 38. At discharge, dosage of prednisolone was 7.5 mg twice a day, of mycophenolate 390 mg three times a day, and of tacrolimus 1.3 mg also three times a day. For infection prevention, we gave aciclovir, trimethoprim, and the antifungals nystatin and amphotericin B per oral. Currently, the patient is doing well but, during follow-up, the patient developed ventricular arrhythmias and recurrent pericardial effusion and although endomyocardial biopsy was without signs of rejection, we treated with prednisolone.

## Background

Kawasaki disease is a rare syndrome of unknown etiology associated with immunological reactions in arteries resulting in vasculitis (1). Although rare, involvement of coronary arteries may occur with dilatation of the coronary arteries and even coronary aneurysms. Interestingly, the coronary aneurysms often regress within the first 2 years. We present the case of a toddler who suffered recurrent myocardial ischemia and myocardial infarction 4 months after diagnosis of Kawasaki disease. He underwent successful heart transplantation.

## Discussion

The first cardiac transplantation in cardiac Kawasaki disease was reported by Travaline and colleagues in 1991. A 14-year-old boy had suffered myocardial infarction 10 weeks after diagnosis of Kawasaki disease, displaying giant coronary artery aneurysms (2). Checchia et al. collected data of pediatric heart transplantation in Kawasaki disease. In 1997, they identified 13 children, worldwide, who had undergone cardiac transplantation (3). The results of 10 children showed that myocardial infarction occurred within 18 months after the outbreak/diagnosis of Kawasaki disease and one-third of children suffered of recurrent myocardial infarction. Although coronary artery bypass grafting (CABG) has been reported (4), in the presented case, it was technically not feasible.

In a large series of patients with Kawasaki disease complicated by giant coronary aneurysms, the long-term data analysis revealed the need of multiple coronary interventions, but rate of re-intervention after CABG was significantly lower than after catheter-based interventions (5). However, CABG has to be discussed critically. In the publication of Checchia et al., 4 out of 10 transplanted patients had undergone previous CABG (left internal mammary artery or saphenous vein), but within 1 year, all four patients had to undergo transplantation due to persistently reduced ventricular function (3).

It is essential to mention possible use of assist devices as a bridge to transplantation in case of refractory arrhythmias or cardiac decompensation. Therefore, we had an extracorporeal life support system in PICU ready for use. Additionally, a biventricular assist device (Berlin Heart Excor) was in the operating theater ready for implantation just in case.

## Concluding Remarks

The presented case was unique due to extensive peripheral stenotic coronary involvement. Heart transplantation is indicated in Kawasaki disease patients with (i) severe impaired ventricular function, (ii) multiple and complex stenotic lesions of the coronary arteries, unsuitable for coronary procedure, or (iii) giant coronary artery aneurysms (with or without thrombi) if the morphology is not suitable for coronary procedure.

## Ethics Statement

This report was carried out in accordance with the recommendations of the local Ethics Committee and parents gave informed consent for publication of this case report and accompanying images.

## Author Contributions

TT contributed to the conception and design of the work, to the acquisition, analysis, and interpretation of data for the work, and drafted the work; MS contributed to the conception of the work, to the analysis and interpretation of data for the work, and drafted the work; MG and AB contributed to the design of the work, to the analysis, and interpretation of data for the work; TP, WR, and FS contributed to the conception and design of the work, to interpretation of data for the work, and revised the work. All authors approved final version of the work.

## Conflict of Interest Statement

The authors declare that the research was conducted in the absence of any commercial or financial relationships that could be construed as a potential conflict of interest.

## References

[1] Kawasaki T Kosaki F Okawa S Shigematsu I Yanagawa H A new infantile acute febrile mucocutaneous lymph-node syndrome (MLNS) prevailing in Japan. Pediatrics (1974) 54:271–6. 4153258

[2] Travaline J Hamilton S Ringel R Laschinger J Ziskind A Cardiac transplantation for giant coronary artery aneurysms complicating Kawasaki disease. Am J Cardiol (1991) 68:560–1. 10.1016/0002-9149(91)90801-Q 1872291

[3] Checchia P Pahl E Shaddy R Shulman S. Cardiac transplantation for Kawasaki disease. Pediatrics (1997) 100:695–9. 10.1542/peds.100.4.695 9310527

[4] Kitamura S Kawachi K Oyama C Miyagi Y Morita R Koh Y Severe Kawasaki heart disease treated with an internal mammary artery graft in pediatric patients. A first successful report. J Thorac Cardiovasc Surg (1985) 89:860–6. 3873581

[5] Suda K Iemura M Nishiono H Teramachi Y Koteda Y Kishimoto S Long-term prognosis of patients with Kawasaki disease complicated by giant coronary aneurysms. A single-institution experience. Circulation (2011) 123:1836–42. 10.1161/CIRCULATIONAHA.110.978213 21502578

